# Supplementary material for: Pathohistological Findings after Bilateral Ovariectomy in Mares with Behavioral Problems
Source: Animals (Basel). 2024 Oct 8;14(19):2899. doi: 10.3390/ani14192899 (PMC11475726; doi:10.3390/ani14192899)
Supplement: Supplementary file 1 [file animals-14-02899-s001.zip › animals-3185417-supplementary.pdf]

Figure S1. Western blotting of antibodies (Aromatase, anti-Müllerian hormone, epidermal growth factor receptor and epithelial cadherin) to test for horse cross-reactivity. Blocking Reagent: Roche 11921673001.

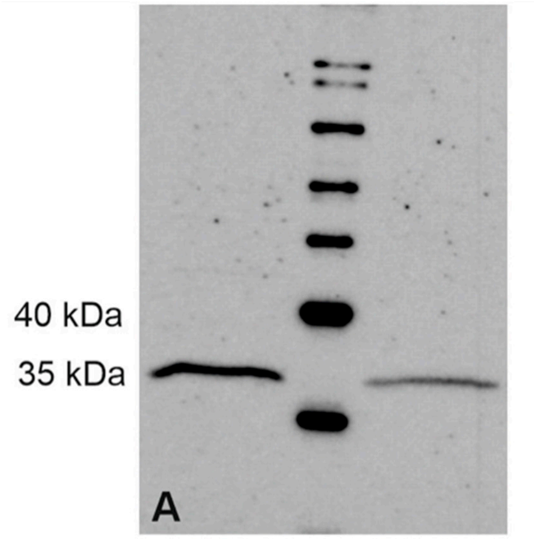

A=aromatase 1: 2000
Gel 10% , Lysis buffer RIPA
Lane 1: positive control human HepG2 cells
Lane 2: molecular weight marker
Lane 3: positive control horse testis

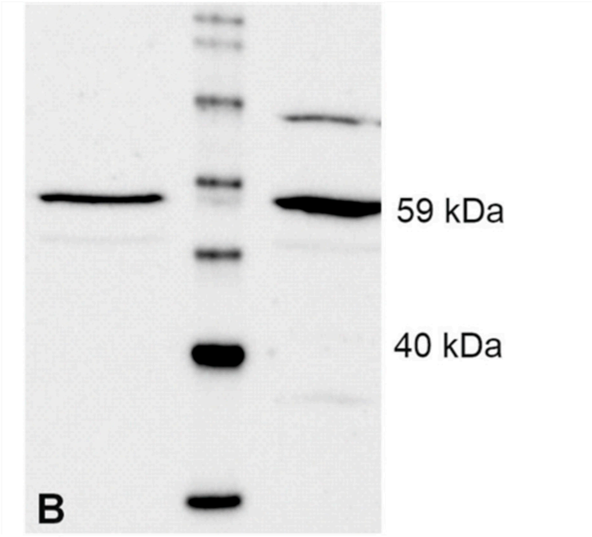

B=anti-Müllerian hormone 1:1000
Gel 10%, Lysis buffer RIPA
Lane 1: positive control human HepG2 cells
Lane 2: molecular weight marker
Lane 3: positive control horse testis

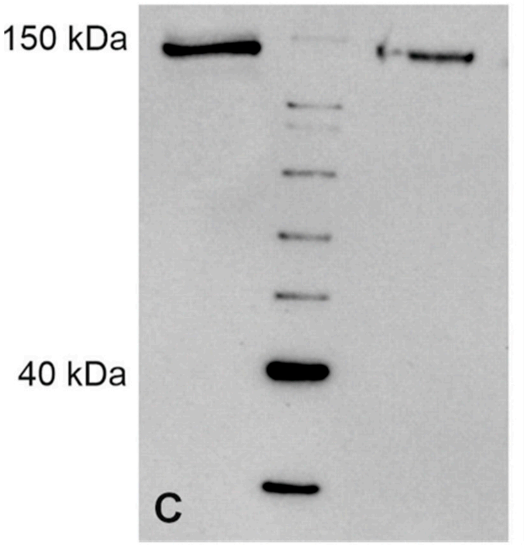

C=epidermal growth factor receptor 1:400
Gel 10% , Lysis buffer RIPA
Lane 1: positive control human A172 cells
Lane 2: molecular weight marker
Lane 3: positive control horse kidney

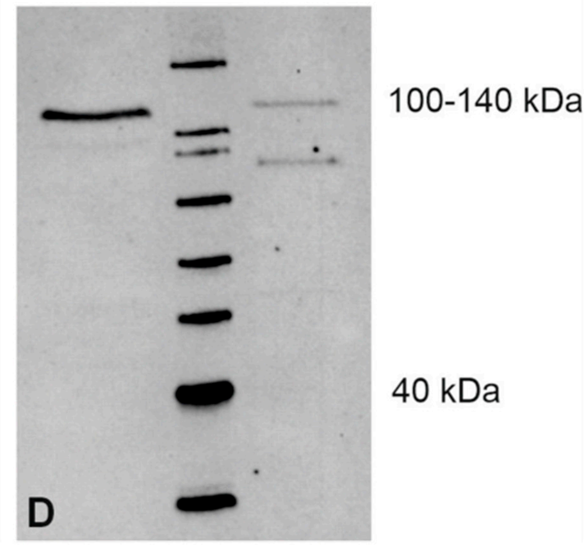

D=epithelial cadherin 1:1000
Gel 12.5% , Lysis buffer Tris Triton
Lane 1: positive control human HepG2 cells
Lane 2: molecular weight marker
Lane 3: positive control horse kidney

Table S1. Summary of findings in bilaterally ovariectomized mares with behavioral problems (bOE) and in mares with granulosa cell tumors (GCT-uOE). Clinical history with behavioral patterns, duration of behavioral problems, and conservative treatment (Altrenogest/GnRH vaccination).

	Case	Age	Clinical History												
			Behavioral Problem from Owner's Perspective										Duration of behavioral Problems (<12 or > 12 months)	Conservative Treatment	
			Moody	Stressed	Unwilling to be ridden	Aggressive towards People	Aggressive towards other Horses	Stallion-like Behavior	Increased Flank Sensitivity	Colic Symptoms	Prolonged or constant estrous Signs	No estrous Signs		GnRH vaccination **(V)**/ oral Altrenogest **(A)**	**Improvement** yes= **+** no= **-**
Bilaterally ovariectomized Mares (**bOE**)	**7**	14	+	+	+	-	-	-	+	+	-	-	<12	**A**	+
	**8**	12	-	+	+	-	-	-	-	-	-	-	<12	**A+V**	+
	**9**	16	+	-	+	-	+	-	-	-	-	-	<12	/	/
	**10**	4	-	-	-	-	-	+	-	-	-	-	>12	/	/
	**15**	12	+	+	+	-	-	-	+	-	-	-	>12	/	/
	**16**	10	-	-	-	-	-	-	-	-	-	-	(Coincidental finding)	/	/
	**23**	9	-	-	-	-	-	-	-	+	+	-	<12	**A**	+
	**25**	11	+	-	-	+	+	-	+	-	-	-	<12	**V**	+
	**29**	16	+	-	-	+	+	-	+	-	+	-	<12	/	/
	**30**	20	-	-	+	+	+	-	-	+	-	-	>12	/	/
	**34**	11	+	+	-	-	-	-	-	-	-	-	<12	/	/
	**35**	12	-	-	+	-	-	-	-	+	-	-	>12	**A+V**	+
	**36**	11	+	-	+	-	-	-	-	-	-	-	<12	/	/
	**38**	14	-	+	-	-	-	-	-	-	+	-	>12	**A**	+
	**39**	16	-	-	-	-	-	-	-	+	-	-	>12	**A**	+
	**40**	8	-	-	+	-	+	-	+	+	-	-	<12	**V**	+
	**46**	16	+	-	-	-	-	-	-	-	+	-	<12	**V**	+
	**47**	14	+	-	-	-	+	-	-	+	-	-	>12	**A**	-
	**48**	15	+	+	+	-	-	-	-	-	-	+	<12	/	/
	**49**	18	-	-	-	-	-	-	-	+	-	-	<12	**A+V**	+
Unilaterally ovariectomized Mares (**GCT-uOE**)	**4**	8	-	+	+	-	-	+	-	-	-	+	>12	/	/
	**6**	7	+	-	+	-	-	-	-	-	-	-	<12	/	/
	**12**	10	-	-	-	-	-	-	-	-	-	-	(Coincidental finding)	/	/
	**24**	18	+	-	-	-	-	+	-	+	-	-	<12	/	/
	**27**	9	-	-	-	-	-	+	-	-	-	+	<12	/	/
	**28**	16	-	-	+	-	-	+	-	-	-	+	<12	/	/
	**32**	16	-	-	-	-	-	+	-	-	+	-	<12	/	/
	**33**	10	-	-	-	-	-	-	-	-	-	+	>12	/	/
	**37**	22	+	-	-	-	-	-	-	-	-	-	<12	/	/
	**44**	16	+	+	-	-	-	+	-	-	-	-	>12	/	/

Table S2. Summary of findings in bilaterally ovariectomized mares with behavioral problems (bOE) and in mares with granulosa cell tumors (GCT-uOE). Clinical examination before surgery with serum hormone concentrations, rectal and ultrasonographical examination, cyclic stage, time of surgery, outcome of surgery, and pathohistological findings in bOE and GCT-uOE.

	Case	Clinical Examination					Time of Surgery (Month)	Outcome of surgery			Pathohistological Evaluation
		Serum Hormone Concentrations: AMH[1] (pmol/L)	Serum Hormone Concentrations: Testosterone[1] (nmol/L)	Size[2] (Rectal palpation)	Structures[3] (Sonography)	Cyclic State[4]		Reoccurrence of behavioral Problems	Persistent estrous Signs after Surgery	Owner's Satisfaction[5]	Histomorphological Abnormalities in bOE[6]/ Special Cells in GCT-uOE[7]
Bilaterally ovariectomized Mares (bOE)	7	9.6	N.m.	Normal	L: mF, R: mF	I	9	-	-	Y	R: Fossa cyst
	8	8.9	N.m.	Normal	L: LF, R: mF	D	10	-	-	Y	No abnormalities
	9	11.6	N.m.	Small	L: mF, R: mF	I	11	-	-	Y	L: Fossa cyst
	10	0.4	Nm.	L: small, R: normal	L: CL, R: mF	D	10	-	-	Y	R: ENCs
	15	5.1	N.m.	Normal	L: LF, R: CL	D	9	-	-	Y	L: AnovF
	16	43.5	N.m.	Normal	L: mF, R: mF	D	9	-	-	Y	No abnormalities
	23	14.3	N.m.	Normal	L: mF, R: LF	E	3	-	-	Y	R: AnovF
	25	30.3	<0.14	Normal	L: LF, R: mF	E	3	-	-	Y	R: Fossa cyst
	29	38.6	0.45	Small	L: mF, R: mF	D	7	-	-	Y	L: ENCs + AnovF, R: ENCs
	30	14.3	<0.14	L: small, R: normal	L: CL, R: mF	D	7	-	-	Y	L: Fossa cyst
	34	15.7	<0.14	L: normal, R: small	L: CL, R: mF	D	10	-	-	Y	L: AnovF
	35	0.8	<0.14	Normal	L: mF, R: mF	I	11	-	-	Y	L+R: Fossa cyst
	36	23.9	<0.14	Normal	L: LF, R: mF	E	10	-	-	Y	R: ENCs + AnovF
	38	11.9	N.m.	Normal	L: mF, R: mF	E	12	-	-	Y	No abnormalities
	39	14.5	<0.14	Normal	L: mF, R: mF	I	12	+	-	N	R: AnovF
	40	8.8	<0.14	L: normal, R: small	L: mF, R: mF	I	1	+	+	N	R: Fossa cyst
	46	1.9	<0.14	Normal	L: LF, R: CL	D	6	-	-	Y	R: Fossa cyst
	47	4.5	<0.14	Normal	L: CL, R: mF	D	6	-	-	Y	No abnormalities
	48	13.2	<0.14	Normal	L: CL, R: CL	D	6	-	-	Y	No abnormalities
	49	5.4.	N.m.	Small	L: mF, R: mF	I	6	+	-	N	No abnormalities
Unilaterally ovariectomized Mares (GCT-uOE)	4	142	0.35	L: extra large*, R: small	L: hc, R: inac	I	7	-	-	Y	GCT
	6	127	0.76	L: large, R: small	L: hc, R: inac	I	9	-	-	Y	GCT; LLC, SLC
	12	143	N.m.	L: extra large*, R: small	L: hc, R: inac	I	6	-	-	Y	GCT; SLC
	24	150	N.m.	L: large, R: small	L: hc, R: inac	I	4	-	-	Y	GCT; LLC (*invasive)
	27	123	0.14	L: large, R: very small	L: hc, R: inac	I	6	-	-	Y	GCT; LLC
	28	142	N.m.	L: small, R: extra large*	L: inac, R: hc	I	6	-	-	Y	GCT; LLC, SLC
	32	150	1.25	L: small, R: large	L: inac, R: hc	I	8	-	-	Y	GCT; LLC, SLC
	33	127	<0.14	L: large, R: very small	L: hc, R: inac	I	9	-	-	Y	GCT; LLC, SLC
	37	150	<0.14	R: large, L: not present	R: hc	I	12	-	+	Y	GCT; SLC
	44	150	0.21	L: small, R: large*	L: inac, R: hc	I	6	-	-	Y	GCT; LLC, SLC

[1] Red labeled values= increased serum concentrations; N.m.=not measured; [2] Size: normal sized=50-80 mm length x 20-40 mm width [43], small sized= < 50 mm length x <20 mm width, large sized= >80 mm length x >40 mm width; extra large*= extra large ovary removed by a 2-step-procedure; L=left ovary, R=right ovary; [3] Structures: mF=multiple follicles, follicles <30 mm diameter; LF=large follicles, follicles >30 mm diameter; CL=corpus luteum; hc=honeycomb-like structure, inac=inactive ovary; L=left ovary, R=right ovary; [4] Cyclic state: E=Estrus stage, D=Diestrus stage, I=Intermediate estrous stage; [5] Owner satisfaction: Y=Yes, N=No; [6] Histomorphological abnormalities in bOE: ENCs=early neoplastic changes, AnovF=anovulatory-like follicle; L=left ovary, R=right ovary; [7] Special cells in diagnosed granulosa cell tumors (GCT) of GCT-uOE: LLC=Leydig-like cells, *invasive= invasive growing LLC; SLC=Sertoli-like cells;

Table S3. Summary of immunohistochemical evaluation in ovaries of bilaterally ovariectomized mares (bOE) and in ovaries of mares with unilaterally removed granulosa cell tumors (GCT-uOE) including Ki-67 (Ki67), anti-Müllerian hormone (AMH), aromatase (AR), epidermal growth factor receptor (EGFR), and calretinin (CAL).

Immunohistochemical Expression of Cell Populations in different Ovarian Structures of bOE and GCT-uOE

**Structures of bOE**	**Cell Population**	*PI grade*[1]	*Intensity of Expression*[2]			
		**Ki67**	**AMH**	**AR**	**EGFR**	**CAL**
Primordial Follicle[3]	Granulosa Cells	0	-	+	++	+
Primary Follicle[3]		0	+	++	++	+
Secondary Follicle[3]		0	+++	++	++	+
Tertiary Follicle[4]	Granulosa Cells	1-3	+++	++	+++	++
	Theca Cells	1	+	+	+	+
Preovulatory Follicle[4]	Granulosa Cells	2-3	+++	+++	+++	++
	Theca interna Cells	1	+	++	++	++
	Theca externa Cells	0	-	+	++	++
Early atretic Follicle[4]	Granulosa Cells	2-3	+++	++	+++	+++
	Theca interna Cells	1	+	++	++	++
	Theca externa Cells	0	-	+	+	+
Late atretic Follicle	Granulosa Cells	0	++	++	+	++
Anovulatory-like Follicle[4]	Granulosa Cells	0-2	+	+	++	++
Early neoplastic Changes	Granulosa Cells	0	+	+++	+++	++
	Leydig-like Cells	0	+	+++	+++	++
Corpus Luteum	Lutein Cells	0	-	+++	+++	+
**Structures of GCT-uOE**						
**Granulosa Cell Tumor**	Granulosa Cells	0-1	++	+/++	+++	++
	Leydig-like Cells	0	+	+++	+++	+++
	Sertoli-like Cells	0	++	+/++	+++	++
	Theca Cells	0	-	-	+	+

[1] The proliferation index (PI) of Ki67 is graded in 0-3: grade 0 for 0-25%, grade 1 for 26-50%, grade 2 for 51-75%, grade 3 for 76-100% stained cells [27]; grade 1-3 means a high PI of >25% stained cells [48]; [2] Intensity of expression: - for negative, + for mild, ++ for moderate and +++ for high expression [31]; [3] Primordial, primary and secondary follicles were summarized as preantral follicles; [4] Tertiary and preovulatory follicles were summarized as antral follicles and together with early atretic and anovulatory-like follicles summarized as large follicles;

Questionnaire SQ1: Owner questionnaire before surgery regarding behavioral patterns, duration of behavioral problems, and conservative treatments.

Owner Questionnaire *before* Ovariectomy

TIERKLINIK STARNBERG
PFERDEZENTRUM

Name of the owner:
Name of the mare:
Age:
Breed:
Ovariectomy: O Unilateral / O Bilateral

1. **Behavioral pattern: does your mare present any of the following behavioral problems?**

- o Unwillingness to be ridden
- o Aggressive behavior towards people
- o Aggressive behavior towards other horses
- o Stallion-like behavior (mounting attempts)
- o Increased flank sensitivity
- o Colic symptoms (regularly/repeated)
- o Constant or prolonged estrous signs
- o No estrous signs
- o Further behavioral disorders: ____

- o My mare does not present any behavioral problem mentioned above.

2. **Duration of behavioral problems: when did you observe behavioral problems in your mare the first time?**

- o < 12 months ago
- o > 12 months ago

3. **Did your mare receive conservative treatment with oral Regumate Equine® before surgery?**

- o Yes
- o No

3.1. **If yes, how was the effect of Regumate Equine® in your mare?**

- o Good effect – symptoms resolved or were markedly improved.
- o No effect
- o Negative effect – symptoms worsened.

4. **Did your mare receive GnRH vaccine before surgery?**

- o Yes
- o No

4.1. **If yes, how was the effect of Regumate Equine® in your mare?**

- Good effect – symptoms resolved or were markedly improved.
- No effect
- Negative effect – symptoms worsened.

Questionnaire SQ2: Telephone questionnaire 6-12 months after surgery regarding behavioral improvement, reoccurrence of behavioral problems, and presence of estrous signs post surgery.

Telephone Questionnaire *after* Ovariectomy

1. **Did your mare show improvement of behavior after ovariectomy?**

- o Yes, completely (all behavioral problems resolved after the surgery)
- o Yes, partly (the following behavioral problems are still present)

  ____

- o No effect was observed after surgery

2. **Did your mare show reoccurrence of behavioral problems?**

- o No
- o Yes, after 1-4 weeks
- o Yes, after 1-6 months
- o Yes, after 6-12 months

3. **Does your mare present any estrous signs post surgery?**

- o Yes, the same as before surgery
- o Yes, but milder than before surgery
- o Yes, but more strongly than before surgery
- o Yes, estrous signs are persistent
- o No, she does not express any estrous signs

4. **If your mare shows estrous signs post surgery, when did they reoccur?**

- o Immediately after surgery (<1 month)
- o After 1-6 months
- o After 6-12 months

5. **Would you recommend the surgery to others?**

- o Yes
- o No
